# Gigacycle fatigue in high strength steels

**DOI:** 10.1080/14686996.2019.1610904

**Published:** 2019-06-21

**Authors:** Yoshiyuki Furuya, Hisashi Hirukawa, Etsuo Takeuchi

**Affiliations:** Research Center for Structural Materials, National Institute for Materials Science, Tsukuba, Japan

**Keywords:** Fatigue, steel, internal fracture, inclusion, crack growth, 10 Engineering and Structural materials, 106 Metallic materials, Mechanical property, Structural materials

## Abstract

This paper reviews the research results to date on gigacycle fatigue caused by internal fractures in high strength steels. Firstly, accelerated fatigue testing was realized using ultrasonic fatigue testing at 20 kHz, which completes 10^9^ cycles in one day, unlike the 3–4 months needed for conventional fatigue testing. Although the frequency effect was anticipated to be problematic, it proved negligible under conditions in which internal fractures occurred. Later, many unique characteristics of internal fractures were elucidated. For example, hydrogen has dramatically greater effects on internal fractures than on conventional surface fractures. Mean stress effects are more serious in titanium alloys than in high strength steels. Size effects were notable in high strength steels. These distinctive characteristics required a unique model to be able to predict gigacycle fatigue strength, which first required elucidation of its mechanisms. To this aim, the author attempted to measure the crack growth rates of small internal cracks using the beach mark method. The results revealed that the crack growth of small internal cracks controls internal fractures. In calculating the crack growth life, however, it was found that the conventional crack growth law overestimates the effects of inclusion size. To rectify this problem, a new model using a new crack growth law was proposed, which predicts more realistic fatigue life curves. As a result, predictions were derived for several grades of high strength steels.

## Graphical abstract

**Figure:**
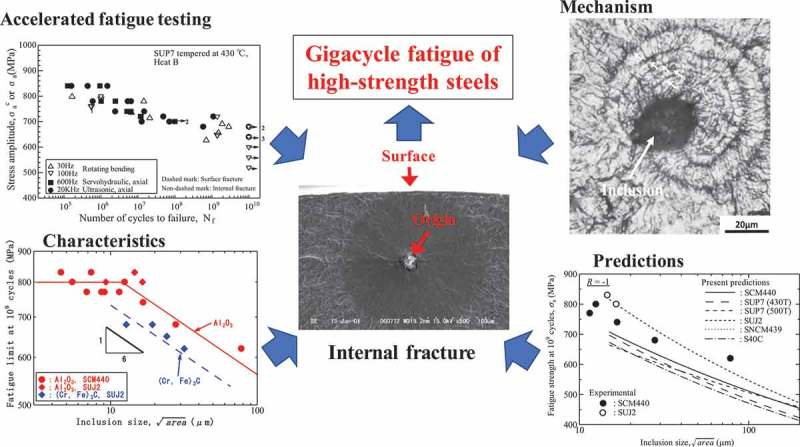

## Introduction

Conventional steels show fatigue limits below which no fatigue failures take place. These fatigue limits can be determined by fatigue testing up to 10^7^ cycles. On the other hand, high strength steels, whose tensile strength exceeds approximately 1200 MPa [1], show very high cycle fatigue (VHCF) failure at over 10^7^ cycles. This disappearance of fatigue limits causes fatigue failures even in the gigacycle region, i.e., above 10^9^ cycles. We therefore call this phenomenon ‘gigacycle fatigue’.

**Figure 1.**
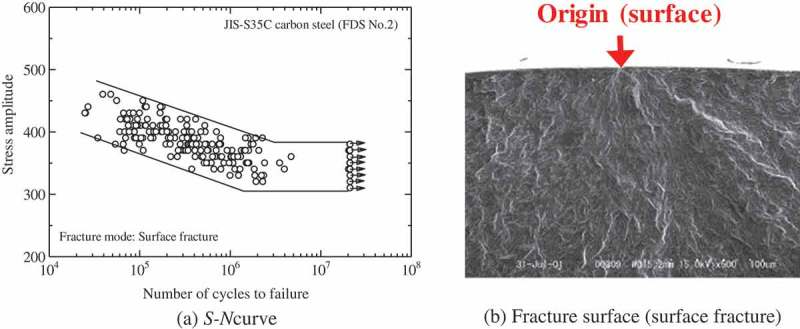
Typical fatigue test results for conventional steel [2].

**Figure 2.**
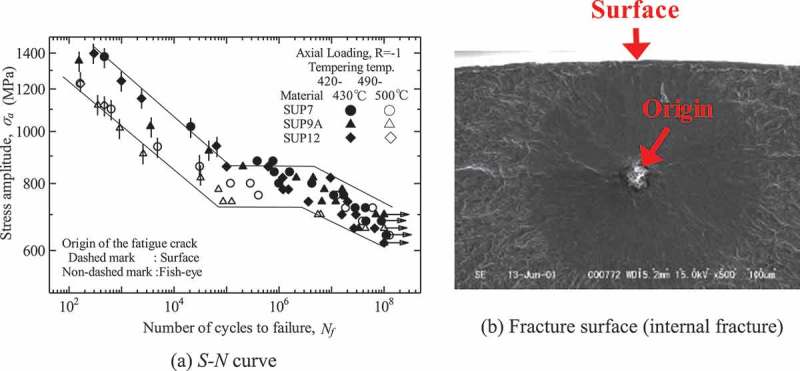
Typical fatigue test results for high strength steel [1].

**Figure 3.**
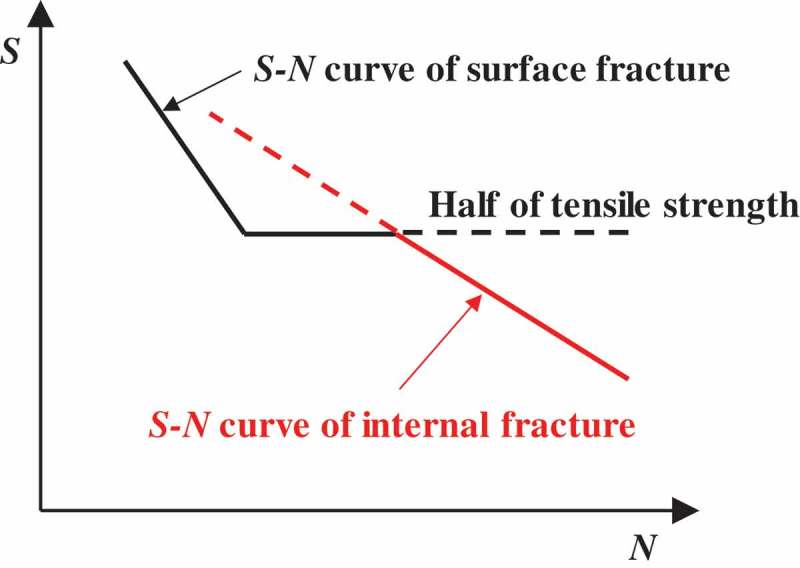
Two-fold *S-N* curve concept.

Figures 1 and 2 show typical fatigue test results for conventional and high strength steels, respectively [1,2]. In conventional steels, the fatigue limit is clear and the fracture modes are surface fractures, i.e., fatigue cracks initiate from surfaces. The fatigue limits are, in general, around half the tensile strength. In contrast, high strength steels develop fatigue failure at above 10^7^ cycles. The fracture mode in the short life region comprises normal surface fracture, whereas internal fractures occur in the VHCF region [3–5]. The internal fractures mostly originate from inclusions. The border of the stress level between the surface and internal fractures is close to the conventional fatigue limit. This means that internal fractures eliminate the conventional fatigue limits. These features can be understood using the two-fold *S-N* curve concept [6,7]. The *S-N* curve is a fatigue life curve which shows the relationship between the applied stress amplitude and the number of cycles to failure. The two-fold *S-N* curve concept illustrated in Figure 3 assumes that the *S-N* curves of internal and surface fractures exist independently, but that a combination of the two *S-N* curves determines their final shape. Internal fractures are thus the key to understanding the gigacycle fatigue seen in high strength steels.

Research on internal fractures first needs an accelerated fatigue testing method, since conventional fatigue testing at 100 Hz takes 3–4 months to reach 10^9^ cycles. Next, gigacycle fatigue tests were conducted on various materials and under various conditions. This step clarified the characteristics of the internal fractures. Finally, the mechanisms of the internal fractures were elucidated to be able to predict gigacycle fatigue strength. This paper reviews the research results to date on the gigacycle fatigue of high strength steels.

## Accelerated fatigue testing using ultrasonic techniques

Ultrasonic fatigue testing [8–12] uses a test frequency of 20 kHz, which is 200 times faster than the conventional 100 Hz. At 20 kHz, the test completes the required 10^9^ cycles in a day. Ultrasonic fatigue testing was standardized in 2017 as WES 1112 by the Japan Welding Engineering Society [13]. Ultrasonic fatigue testing machines are also commercially available.

**Figure 4.**
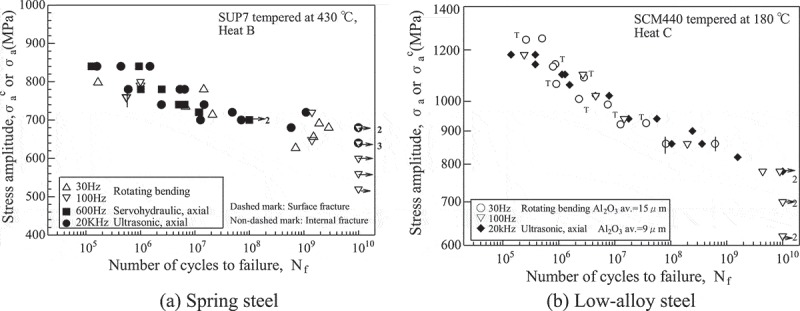
Typical ultrasonic fatigue test results for high strength steels [22,25].

**Figure 5.**
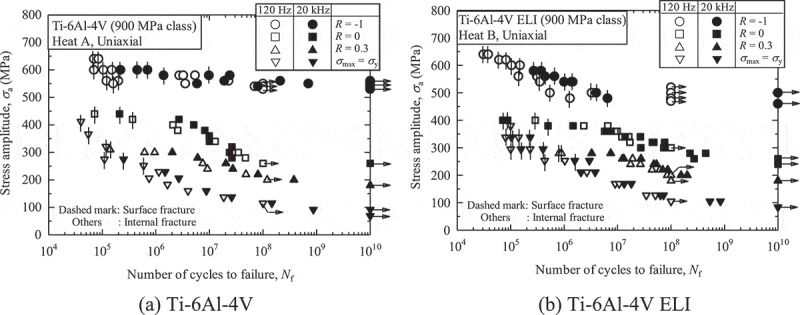
Typical ultrasonic fatigue test results for titanium alloys [27,46].

A point to be noted is the frequency effect. This is the question of whether or not the results of ultrasonic fatigue testing are comparable to those obtained by conventional fatigue testing. Some results have in fact suggested that the frequency effect is not always negligible [14–18]. However, our results indicate that it is negligible in conditions under which internal fractures occur [19–23]. To test for possible frequency effects, we conducted conventional fatigue tests at 100 Hz up to 10^10^ cycles for three years [24], and compared the results with those from ultrasonic fatigue tests. Figure 4 shows typical results. Ultrasonic fatigue testing shows good agreement with conventional fatigue testing. Similar results were also obtained for titanium alloys, as seen in Figure 5 [25–27]. The frequency effect is summarized in the ‘Explanation’ part of WES 1112 [13]. These results indicate that ultrasonic fatigue testing can be used successfully to carry out research on the gigacycle fatigue caused by internal fractures. We thus applied accelerated (ultrasonic) fatigue testing.

Ultrasonic fatigue testing is also effective to inspect for inclusions [11,28–30]. Internal fracture surfaces reveal inclusions at the fracture origin, and an extreme value distribution can be obtained by measuring the inclusion sizes. Ultrasonic fatigue testing creates internal fracture surfaces very rapidly. Other applications of ultrasonic fatigue testing have also been reported [31–37], including our work on high-temperature ultrasonic fatigue testing [38,39].

## The characteristics of internal fractures

**Figure 6.**
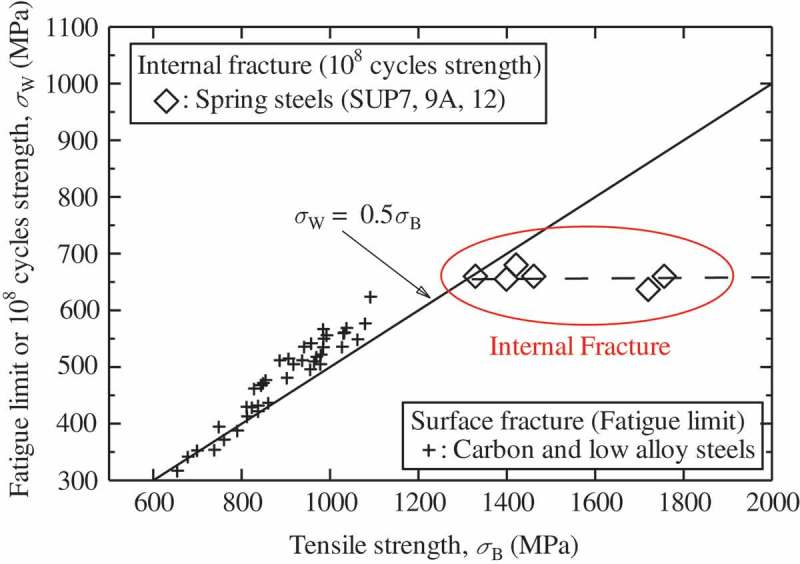
Effect of tensile strength on fatigue strength [1].

**Figure 7.**
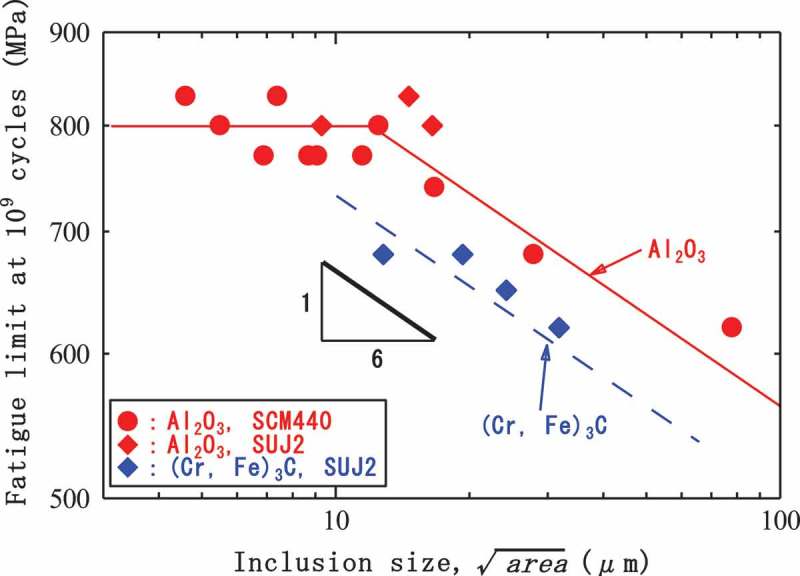
10^9^ cycles fatigue strength as a function of inclusion size [40]. Reprinted by permission from Springer Nature, Metallurgical and Materials Transactions A, Gigacycle Fatigue Properties of High-Strength Steels According to Inclusion and ODA Sizes, Y. Furuya, H. Hirukawa, T. Kimura et al, 2007.

Conventional surface fractures show fatigue limits, while internal fractures do not. Internal fractures also show many other characteristics that distinguish them from conventional surface fractures. Figure 6 shows the effect of tensile strength on fatigue strength. Although the fatigue strengths of the surface fractures are closely dependent on the material’s tensile strength, the effect of tensile strength on internal fractures is very minor. However, internal fractures are affected by the sizes of inclusions at the fracture origins. Figure 7 shows experimentally determined 10^9^-cycle fatigue strengths as a function of inclusion size [40]. The fatigue strengths of the internal fractures decrease with increasing inclusion size. These results show that the controlling factors are different for internal and surface fractures.

**Figure 8.**
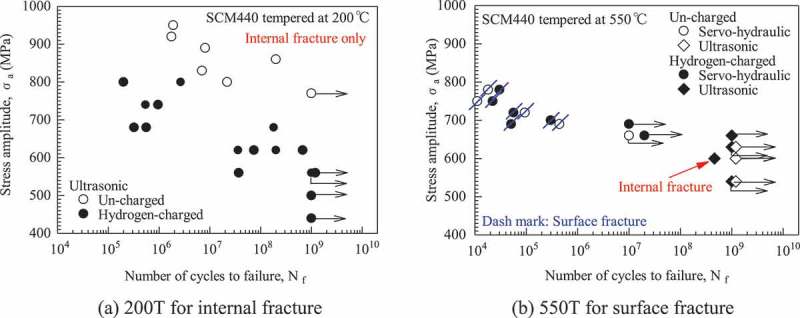
Typical fatigue test results for hydrogen-charged specimens of low-alloy steels [42]. Reproduced by permission of the Iron and Steel Institute of Japan, Hisashi Hirukawa, Yoshiyuki Furuya, Gigacycle fatigue properties of hydrogen charged high strength steels. Tetsu to Hagane, 99, 494–501, Copyright 2013.

Differences are also reported in hydrogen effects [41–43], mean stress effects [44–46] and size effects [47–51]. Figure 8 shows typical fatigue test results for hydrogen-charged specimens of low-alloy steels [42]. The low-alloy steel used was a double-melted (VAR) version of very high cleanliness. The 200T specimens are high-hardness types that show only internal fractures. The 550T specimens are of low hardness and in most cases show surface fractures. As seen in Figure 8(a), the fatigue strength drops significantly in the hydrogen-charged specimens once the specimens develop internal fractures. In contrast, the loss of fatigue strength is very minor with surface fractures. These observations show that the effects of hydrogen on internal fractures are dramatically greater than its effects on surface fractures. This difference occasionally causes a fracture mode change from surface fractures to internal fractures. For example, a hydrogen-charged specimen of 550T developed an internal fracture, as seen in Figure 8(b), whereas 550T specimens never develop such fractures in an un-charged condition. This fracture mode change can be understood based on the two-fold *S-N* curve concept, i.e., only the *S-N* curve of the internal fracture drops. We need to pay close attention to this change in fracture mode, since it could occur in real materials [43].

**Figure 9.**
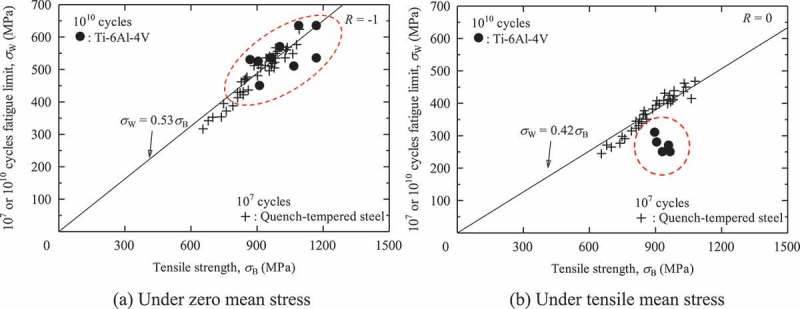
Comparisons of fatigue strength between Ti-6Al-4V alloys and steels [27, 46].

**Figure 10.**
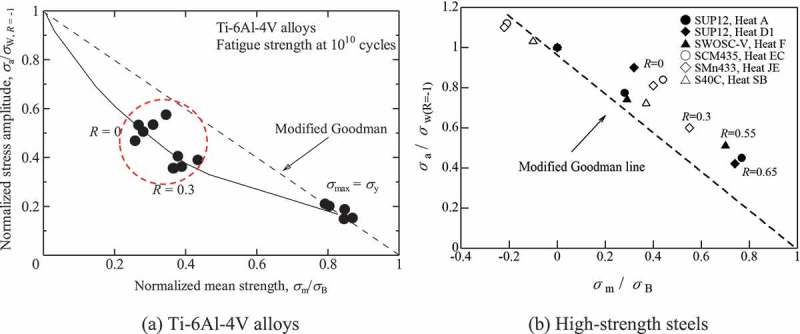
Endurance limit diagrams [27,46,53]. Figure 10(b) reprinted from Materials & Design, 32(3), Yoshiyuki Furuya, Takayuki Abe, Effect of mean stress on fatigue properties of 1800 MPa-class spring steels, 1101–1107, Copyright (2011), with permission from Elsevier.

The mean stress effects on internal fractures are more serious in titanium alloys than in high strength steels. As seen in Figure 5, Ti-6Al-4V alloys are more prone to internal fracturing under tensile mean stress conditions, i.e., under stress ratios of *R* ≥ 0. These internal fractures under tensile mean stress degrade the gigacycle fatigue strength of Ti-6Al-4V alloys [46]. Figure 9 shows comparisons of fatigue strength between Ti-6Al-4V alloys and steels. The fatigue strengths of Ti-6Al-4V alloys are comparable to those of quench-tempered steel under zero mean stress conditions. Under tensile mean stress conditions, however, the Ti-6A-4V alloys show evidently lower fatigue strength. This large mean stress effect causes a serious problem. Figure 10 shows endurance limit diagrams for Ti-6Al-4V alloys and for high strength steels. The Ti-6Al-4V alloys show lower fatigue strengths than their modified Goodman lines. In general, modified Goodman lines provide predictions with a wide safety margin, so the actual fatigue strengths are higher than those suggested by the modified Goodman lines. In Ti-6Al-4V alloys, however, the modified Goodman lines stray into the danger area. This phenomenon is seen uniquely in gigacycle fatigue since conventional 10^7^ cycles fatigue strengths show good agreements with the modified Goodman lines [27]. Although the reason remains unknown, twinning may be a factor. Ono et al. reported twin-induced fatigue cracks in Ti-5Al-2.5Sn ELI alloy tested at *R* = 0.01 at cryogenic temperatures, which were detrimental to the fatigue strength [52]. The twinning occurs under severe conditions such as at cryogenic temperatures, while the gigacycle may be a kind of the severe conditions. We should, therefore, be very careful about the mean stress effects on gigacycle fatigue strength when using titanium alloys. On the other hand, these trends are not observed in high strength steels [53]. Figure 10(b) indicates that the fatigue strengths of high strength steels are higher than the modified Goodman lines. The modified Goodman lines can, therefore, be used safely with high strength steels.

**Figure 11.**
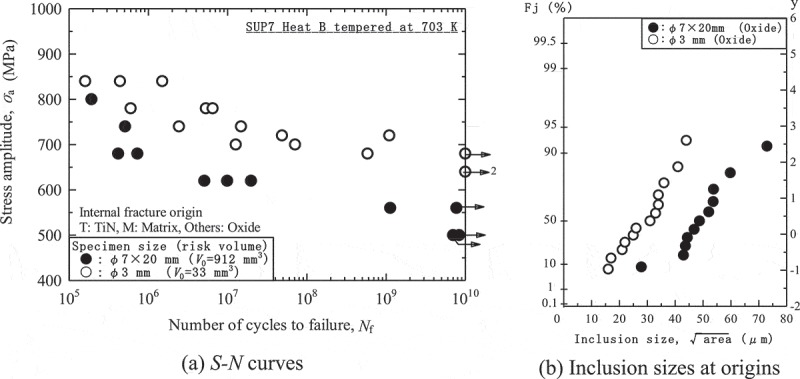
Size effects in gigacycle fatigue of high strength steels [50].  Reprinted from Materials Science and Engineering: A, 528, Yoshiyuki Furuya, Notable size effects on very high cycle fatigue properties of high strength steel, 5234–5240, Copyright (2011), with permission from Elsevier.

However, size effects are notable in high strength steels. Figure 11 shows typical fatigue test results for size effects in high strength steels [50]. The fatigue tests were conducted on a spring steel using two specimen sizes. One was a 3 mm-diameter specimen without straight sections. The other was a 7 mm-diameter specimen with 20-mm straight sections. The 7 mm-diameter specimens had distinctly lower fatigue strength. The degradation of their fatigue strength was caused by larger inclusions appearing at the internal fracture origins, as seen in Figure 11(b). The mechanism is therefore as follows. Large specimens contain more inclusions, increasing the probability of their containing large inclusions. This leads to large inclusions appearing at the internal fracture origins, resulting in degradation of fatigue strength. These results mean that using large specimens is recommended in fatigue tests of high strength steels.

In summary, the characteristics of internal fractures are considerably different from those of conventional surface fractures, and these size effects pose a very serious problem. Figure 11(a) indicates that *S-N* curves depend on specimen size. Accordingly, these *S-N* curves cannot be applied when designing actual components, since the component sizes are different from the specimen sizes. This means that fatigue tests would have to be conducted on each component: an unrealistic and prohibitively expensive approach. To overcome this problem, the fatigue strength should be evaluated using a strategy that differs from the conventional *S-N* curve approach. Fortunately, this basic strategy already exists: it was proposed by Murakami et al. [3,54,55]. This strategy comprises two steps.

1. Estimation of the maximal inclusion size contained in the steel or component.
2. Estimation of the fatigue strength as a function of the maximal inclusion size.

The first step uses extreme value analysis [56,57]. It has already been shown to provide good estimations [50], so this step is already completed from the viewpoint of academic research. On the other hand, the second step needs further study. Although the Murakami equation is well established [3], it has room for improvement in its applicability to gigacycle fatigue. In these academic studies, a mechanism needs to be clarified that accurately describes internal fractures, and a model must be created that can predict the gigacycle fatigue strength of high strength steels. The present state of these academic studies is reviewed in the following sections.

## Mechanisms of internal fracture

Fatigue failure is caused by cracks. Small cracks initiate and grow into critical sizes that cause catastrophic failure. At around the stress level of the fatigue limit, non-propagating cracks are present, i.e., small cracks initiate, but they stop growing. Fatigue failure thus has three processes, which are crack initiation, crack growth and non-propagating cracks. This mechanism is also seen with internal fractures. A key point that should be clarified here is which process controls the internal fracture. This clarification is necessary to make a model for predicting gigacycle fatigue strength, since each process requires its own concept on which to base a model.

Some reports attribute gigacycle fatigue failure to crack initiation [58–60]. Even if the crack propagation rate, i.e., crack propagation length per cycle, is as small as one lattice length, gigacycle fatigue testing results in cracks that are several hundred millimeters long, far exceeding the diameter of the fatigue test specimens. For this reason, some researchers assert that crack initiation is dominant. In this case, however, the inclusion size is difficult to take into account. A key mechanical factor that affects crack initiation is the stress concentration factor, which depends chiefly on the shape of the inclusion, regardless of its size. Thus, a mechanism derived from crack initiation is unlikely to be usable when creating a model.

Using fracture mechanics is one way of taking the inclusion size into account. The stress intensity factor used in fracture mechanics needs to include crack size or inclusion size in its formulation. This method assumes that a non-propagating crack or crack propagation controls internal facture. The Murakami equation [3] is typical in that it assumes a non-propagating crack to be the controlling factor. Although it leads to fatigue limits that disappear with internal fractures, Murakami et al. claim that the disappearance of the fatigue limit can be explained by the presence of optically dark areas (ODAs) [61,62]. An ODA is a special feature of fracture surfaces at around the internal fracture origins, which are observed only when the specimens fail in the VHCF region. This has prompted numerous researchers to investigate ODAs [63–67]. However, the nature of ODAs is still unclear, and a growth law has yet to be established for them. A reasonable suggestion is that ODAs are formed due to contact of the fracture surfaces in vacuum [67]. This could explain why ODAs are solely visible for loads under *R* = −1 [68]. This suggestion implies that the ODAs are trace of crack growth.

Another approach using fracture mechanics assumes crack propagation to be the controlling factor. The crack propagation life is estimated under the assumption that the crack propagation life is nearly equal to the total life. Tanaka and Akiniwa were the first to apply this methodology to internal fractures [69]. Although it relies on internal crack propagation rates, which are very difficult to measure, Tanaka and Akiniwa proposed a method for reverse-calculating the internal crack growth rates using conventional fatigue test results which measure only total fatigue lives and the inclusion sizes of the crack origins. A simpler version of this reverse calculation was then proposed by Omata [70]. This concept is called the Tanaka-Akiniwa model in this paper. A problem with the Tanaka-Akiniwa model is the validity of the calculated internal crack growth rates. As suggested in the discussion on crack initiation being dominant, extremely slow crack growth rates, which are much smaller than the lattice length, are yielded when the Tanaka-Akiniwa model is applied to gigacycle fatigue [71–73]. The Tanaka-Akiniwa model, therefore, needs experimental confirmation of the existence of this extremely slow crack growth.

**Figure 12.**
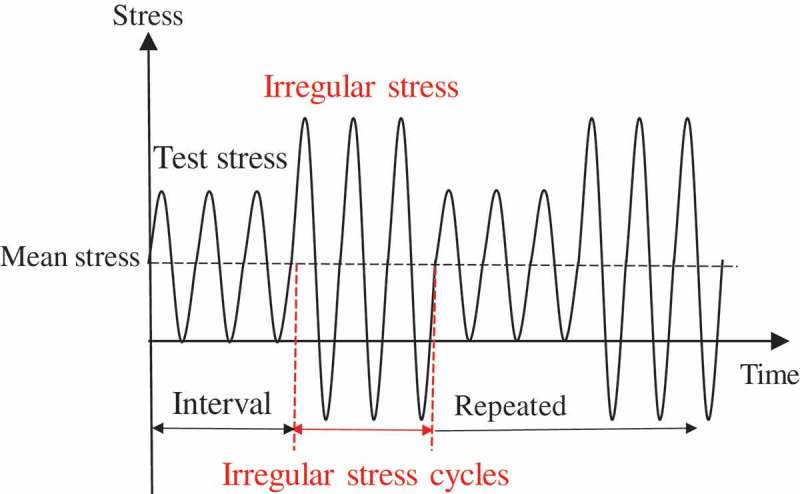
Typical waveform used in the repeated two-step fatigue tests.  Reprinted from Materials Science and Engineering: A, 528, Yoshiyuki Furuya, Notable size effects on very high cycle fatigue properties of high strength steel, 5234–5240, Copyright (2011), with permission from Elsevier.

To seek answers to these questions, the author tried to measure the crack growth rates of small internal cracks. Internal cracks, however, unlike normal surface cracks, cannot be observed directly. The author, therefore, established a method of visualizing small internal fatigue crack growth using beach marks [74]. Beach marks are a feature of a fracture surface that results from irregular loading during fatigue [75]. Controlled beach marks can be created by repeated two-step fatigue tests. Figure 12 shows a typical waveform used in repeated two-step tests. Controlled irregular stresses are inserted at controlled intervals. These controlled irregular stresses create the controlled beach marks that reveal the traces of crack growth.

**Figure 13.**
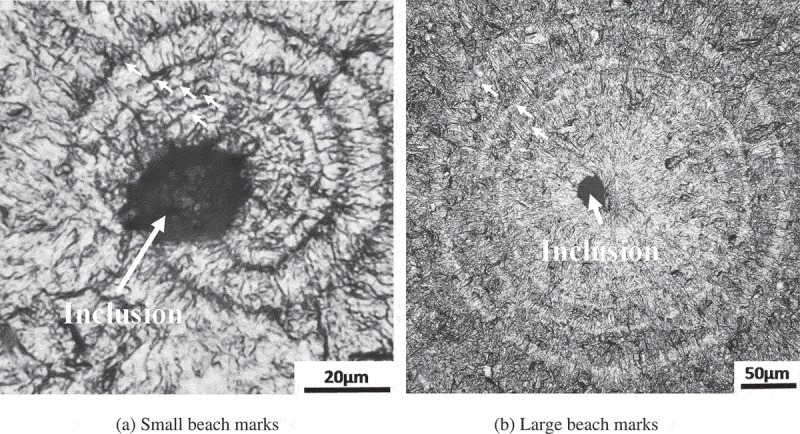
Typical beach marks observed in ref [76]. Reprinted from Materials Science and Engineering: A, 678, Yoshiyuki Furuya, Small internal fatigue crack growth rate measured by beach marks, 260–266, Copyright (2016), with permission from Elsevier.

Figure 13 shows typical beach marks observed in ref [76]. There were two types: one was small, suggesting a small internal crack just after crack initiation. The other was large, indicating an internal crack in the final stage of fatigue life. The key point here is the shape of the internal crack. Although an internal crack in the final stage reveals a circular and symmetric shape, a small internal crack takes the form of an asymmetric half-ellipse. The extremely slow crack growth of the internal cracks is attributable to the asymmetric shape of the small internal crack. Although two-dimensional modeling is possible for circular internal cracks, small asymmetric internal cracks require three-dimensional modeling. On the other hand, the calculation of crack propagation life normally assumes two-dimensional modeling. If we presuppose that the average crack growth rate of an asymmetric crack corresponds to the crack propagation rate in two-dimensional modeling, the two-dimensional crack propagation rate could be very slow: even if the local crack growth exceeds the lattice length, the average crack growth rate could be smaller than the lattice length. This is the mechanism by which extremely slow crack growth rates can be calculated.

**Figure 14.**
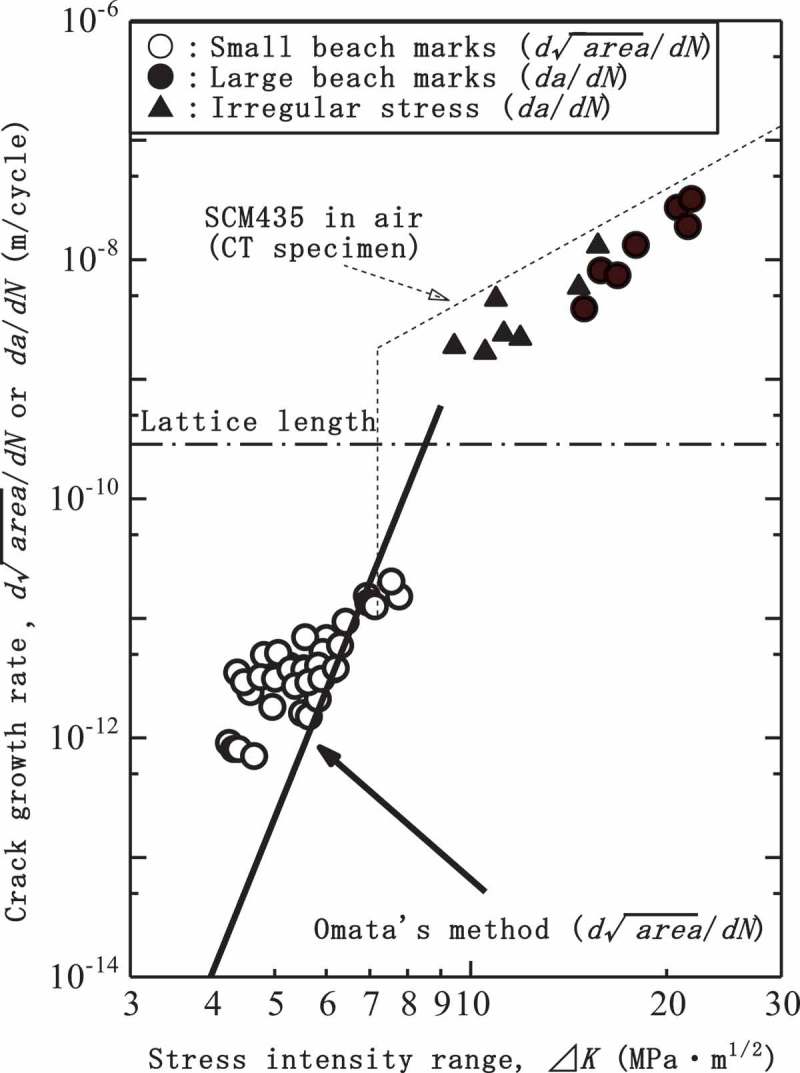
Internal crack growth rates measured by beach marks [76]. Reprinted from Materials Science and Engineering: A, 678, Yoshiyuki Furuya, Small internal fatigue crack growth rate measured by beach marks, 260–266, Copyright (2016), with permission from Elsevier.

Figure 14 shows internal crack growth rates measured using beach marks. The open and solid marks respectively show the crack growth rates of small and normal internal cracks. The small internal cracks reveal extremely slow-growing rates that are much smaller than the lattice length. On the other hand, the growth rates of the normal internal cracks are larger than the lattice length and close to the conventional crack growth rate indicated by the dotted line. In terms of *∆K*, the border between the small and normal internal cracks is close to the threshold stress intensity range *∆K*_th_ for conventional large cracks. The small internal cracks grow under *∆K* conditions below *∆K*_th_. The area of the small internal cracks can thus be regarded as an ODA. These growth behaviors of internal cracks are exactly what is proposed in the Tanaka-Akiniwa model. These results thus support the validity of the Tanaka-Akiniwa model. In other words, these results mean that the crack growth of the small internal cracks controls the internal fracture. Note that clearly observed beach marks were fewer than irregular stresses [74]. Resolution of the beach marks was in fact not so high as to identify the cycle number of the crack initiation, while it was also interpreted that the crack initiation took a certain number of cycles. The crack initiation life causes differences between measured and calculated crack growth rates since the calculation ignores the crack initiation life. In Figure 14, a solid and bold line indicates the crack growth rate calculated by the Omata’s method, which was slower than the measurements. These differences potentially cause errors when the crack growth is assumed to be the controlling factor.

## Models for predicting gigacycle fatigue strength

When the crack growth is the controlling factor, the fatigue life is nearly equal to the crack growth life that can be calculated from the crack growth rates. The crack growth rates of the internal cracks are calculated in reverse using conventional fatigue test results, as proposed in the Tanaka-Akiniwa model [69]. A simpler version of this reverse calculation, proposed by Omata [70], is as follows.

The calculation starts with the Paris law, a conventional fatigue crack growth law.

(1) $$\frac{da}{dN}=C{\left(\Delta K\right)}^{m}$$

*C* and *m* are constants that determine the crack growth properties. *a* is crack length and *N* is cycle number, so *da/dN* is the crack growth rate. The stress intensity range, *∆K*, is evaluated for internal cracks using the following equation [3].

(2) $$\Delta K=0.5\Delta \sigma \sqrt{\pi \sqrt{area}},\;\Delta \sigma =2\sigma_{a}$$

where $\sqrt{area}$ is the crack size expressed as the square root of the projected area, and *σ*_a_ is half the stress range. By integrating Equation (1) in the range of $\sqrt{area}$ from an inclusion size $\sqrt{area}$_inc_ to infinity, the following equation is obtained.

(3) $${\left(\Delta K_{inc}\right)}^{m}\left(\frac{N_{f}}{{\sqrt{area}}_{inc}}\right)=\frac{2}{C\left(m-2\right)}$$

where *∆K*_inc_ is the stress intensity range at the inclusion size and *N*_f_ is the fatigue life. As understood from Equation (3), *C* and *m* are determined by fitting the relationship between *∆K*_inc_ and *N*_f_/$\sqrt{area}$_inc_, which can be calculated from conventional fatigue test results. Equation (3) is, moreover, transformed into the following equation to predict the fatigue strength.

(4) $$\sigma_{a}=\frac{1}{\sqrt{\pi }}{\left(\frac{2}{C\left(m-2\right)}\right)}^{\frac{1}{m}}\times {\left(N_{f}\right)}^{-\frac{1}{m}}\times {\left({\sqrt{area}}_{inc}\right)}^{\left(\frac{1}{m}-\frac{1}{2}\right)}$$

**Figure 15.**
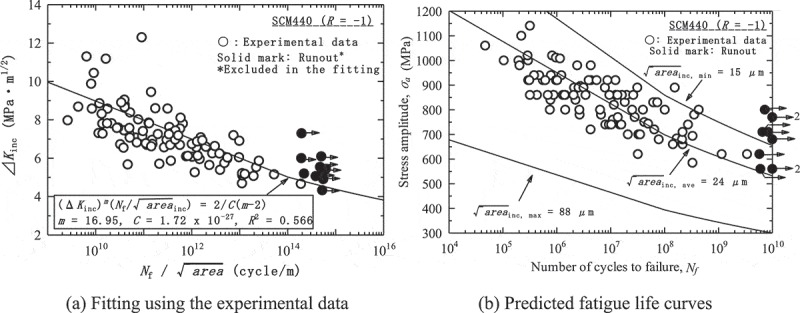
Example of predictions based on the Tanaka-Akiniwa model [77]. Reprinted from Materials Science and Engineering: A, 743, Yoshiyuki Furuya, A new model for predicting the gigacycle fatigue strength of high strength steels, 445–452, Copyright (2019), with permission from Elsevier.

Figure 15 shows an example of the fitting and the predicted fatigue life curves. The fatigue life curves are predicted for minimum, average and maximum inclusion sizes in the experimental data, which are 15, 24 and 84 µm, respectively. When the predictions are compared with the experimental data, the predicted fatigue life curves for minimum and average inclusion sizes are fairly close. A problem is the predicted fatigue life curve for the maximum inclusion size, which is far below the experimental data. The fatigue strength at 10^10^ cycles approaches 300 MPa, which is extremely unlikely. The Tanaka-Akiniwa model thus overestimates the effects of inclusion sizes.

To correct this problem, a new model has been proposed [76,77]. Its key feature is to use the following new crack growth law instead of the conventional Paris law.

(5) $$\frac{d\sqrt{area}}{dN}=C{\left(\Delta K\cdot {\sqrt{area}}^{\alpha }\right)}^{m}$$

where *α* is a new constant. Equation (5) states that the crack growth rate depends not only on Δ*K* but also on the crack size. This idea is acceptable, since it has already been pointed out that the threshold stress intensity range Δ*K*_th_ depends on the crack size for small cracks [3]. To match the crack growth rate to Δ*K*_th_ of the small cracks, it is necessary for the crack growth rate to also depend on the crack size.

**Figure 16.**
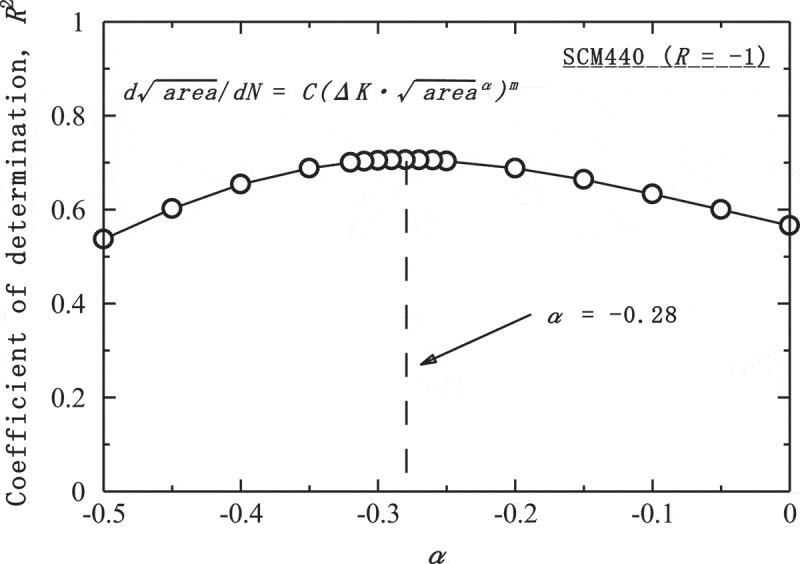
*R* ^2^-values for various values of *α* in the new model [77]. Reprinted from Materials Science and Engineering: A, 743, Yoshiyuki Furuya, A new model for predicting the gigacycle fatigue strength of high strength steels, 445–452, Copyright (2019), with permission from Elsevier.

The new crack growth law is applied only to small internal cracks. The growth areas of the small internal cracks correspond to ODAs whose sizes are double or triple the inclusion sizes [20,61,62]. When integrating Equation (5), therefore, the appropriate integral range is from $\sqrt{area}$_inc_ to 2$\sqrt{area}$_inc_. The following equation is then obtained:

(6) $${\left(\Delta K_{inc}\cdot {\sqrt{area}}_{inc}^{\alpha }\right)}^{m}\left(\frac{N_{f}}{{\sqrt{area}}_{inc}}\right)=\frac{2^{1-m\left(\frac{1}{2}+\alpha \right)}-1}{C\left(1-m\left(\frac{1}{2}+\alpha \right)\right)}=D$$

where *D* is a constant that simplifies the right side of Equation (6). Constants are basically determined by fitting the relationship between $\Delta K_{inc}\cdot {\sqrt{area}}_{inc}^{\alpha }$ and *N*_f_/$\sqrt{area}$_inc_. The problem is then how to determine the new constant *α*. To solve this problem, *R* ^2^-values (coefficient of determination) are evaluated for various values of *α* as shown in Figure 16. The *R* ^2^ values then reveal a peak at a certain value of *α*. The *R* ^2^ values reveal the goodness of fit, i.e., the closer to 1 the *R* ^2^-values is, the better the fit. The value of *α* can therefore be determined using this peak. The Tanaka-Akiniwa model corresponds to the condition of *α* = 0, so the *R* ^2^-values are improved from 0.566 to 0.706 using the new model. By transforming Equation (6), the following equation to predict the fatigue strength is obtained.

(7) $$\begin{array}{rl} & \sigma_{a}=\frac{1}{\sqrt{\pi }}{\left(D\right)}^{\frac{1}{m}}\times {\left(N_{f}\right)}^{-\frac{1}{m}}\times {\left({\sqrt{area}}_{inc}\right)}^{\left(\frac{1}{m}-\frac{1}{2}-\alpha \right)},\\ & D=\frac{2^{1-m\left(\frac{1}{2}+\alpha \right)}-1}{C\left(1-m\left(\frac{1}{2}+\alpha \right)\right)} \end{array}$$

**Figure 17.**
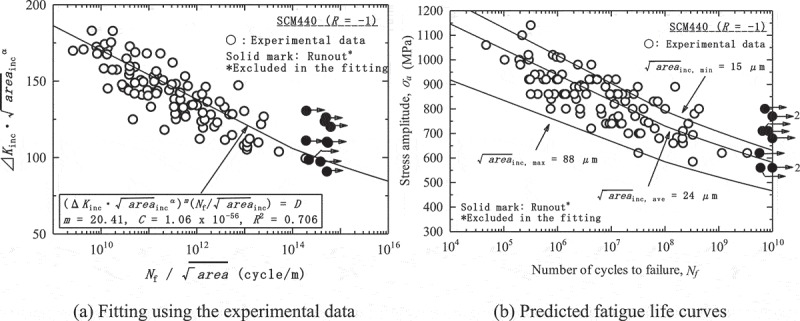
Predictions based on the new model using the same data as in Figure 15 [77]. Reprinted from Materials Science and Engineering: A, 743, Yoshiyuki Furuya, A new model for predicting the gigacycle fatigue strength of high strength steels, 445–452, Copyright (2019), with permission from Elsevier.

Figure 17 shows the predictions based on the new model using the same experimental data as in Figure 15. Although some data points fall outside the predicted band, the fatigue life curve for maximum inclusion size is considerably improved over that of the Tanaka-Akiniwa model. The predictions based on the new model are thus more realistic.

The predictions based on the new model are derived for several grades of high strength steels [77]. Equation (7) is then rewritten in the following form to improve its usability.

(8) $$\sigma_{a}=A1\times {\left(N_{f}\right)}^{a2}\times {\left({\sqrt{area}}_{inc}\right)}^{a3}$$

**Figure 18.**
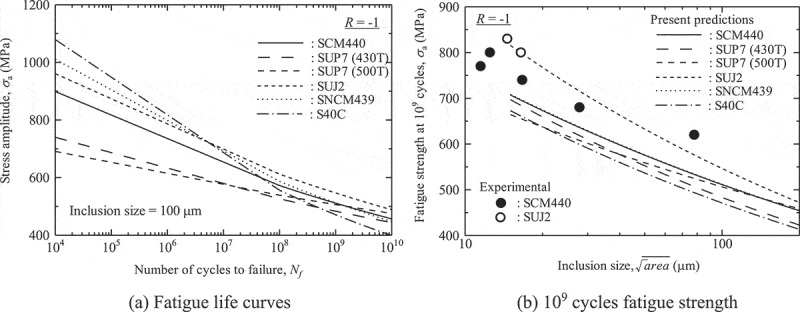
Predicted fatigue strength [77]. Reprinted from Materials Science and Engineering: A, 743, Yoshiyuki Furuya, A new model for predicting the gigacycle fatigue strength of high strength steels, 445–452, Copyright (2019), with permission from Elsevier.

**Figure 19.**
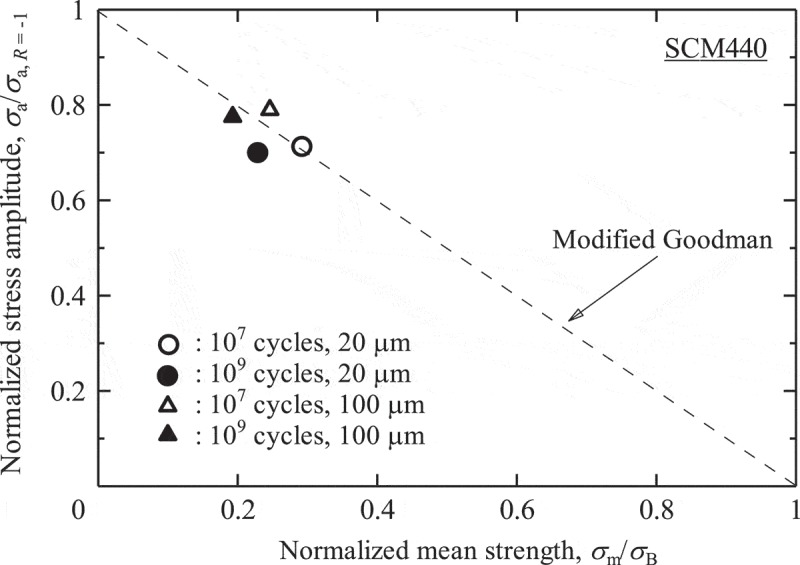
Endurance limit diagram with the predictions in ref [77]. Reprinted from Materials Science and Engineering: A, 743, Yoshiyuki Furuya, A new model for predicting the gigacycle fatigue strength of high strength steels, 445–452, Copyright (2019), with permission from Elsevier.

The constants of *A*1, *a*2 and *a*3 are calculated from those of *C, m* and *α*. Table 1 shows the calculated values. These are the latest predictions for the gigacycle fatigue strengths of high strength steels. Figure 18 shows them. As shown in Figure 18(a), the differences between steel grades are major in the short life region, whereas they are minor in the long life region. Figure 18(b) shows that the decrease in fatigue strengths becomes less steep as a function of rising inclusion size. As a result, even for 100-µm inclusions, the 10^9^ cycle fatigue strengths are about 500 MPa. Figure 19 is an endurance limit diagram. The stress ratios for JIS-SCM440 in Table 1 is not only *R* = −1 but also *R* = 0, so the endurance limit diagram can be drawn for this steel grade. The predicted fatigue strengths are very close to the modified Goodman line. Therefore, the mean stress effects can be safely evaluated using the modified Goodman line, without it entering the danger area as seen in Ti-6Al-4V alloys.

**Table 1.** Calculated values of *A*1, *a*2 and *a*3 for Equation (8), reproduced with permission from [77].

Steel	Vickers hardness	Stress ratio	Number of data points	Constants		
				*A*1	*a*2	*a*3
JIS-SCM440 (Low-alloy steel)	611	*R* = −1	95	292.2	−0.049	−0.171
		*R* = 0	20	443.8	−0.053	−0.107
JIS-SUP7-430T (Spring steel)	527	*R* = −1	42	175.9	−0.037	−0.193
JIS-SUP7-500T (Spring steel)	438	*R* = −1	29	237.4	−0.027	−0.143
JIS-SUJ2 (Bearing steel)	753	*R* = −1	43	216.1	−0.049	−0.211
JIS-SNCM439 (Low-alloy steel)	598	*R* = −1	20	360.3	−0.059	−0.171
JIS-S40C (Carbon steel)	585	*R* = −1	24	370.8	−0.072	−0.188

The predictions in ref [77]. are, however, dependent on the grade of steel employed. This means that other steel grades, which are not listed in Table 1, require their own predictions derived after additional gigacycle fatigue testing. Namely, more gigacycle fatigue tests are necessary to cover more steel grades. However, the gigacycle fatigue tests are easily conducted by using the ultrasonic fatigue testing. Note that the predictions in ref [77]. cover only globular oxide-type inclusions that are classified as type D in the ASTM standard [78], whereas there are other types of inclusions such as sulfides (type A), rows of oxides (type B) and silicates (type C). More research will be needed in future to encompass these other types of inclusions. Moreover, the gigacycle fatigue should be studied also on the other metallic materials. A major point discussed in the gigacycle fatigue was how the internal defects affect the fatigue properties. From this point of view, metallic alloys processed by additive manufacturing (AM) are the typical material in which fatigue properties are affected by the defects [79].

## Summary

This paper reviewed the research results on gigacycle fatigue caused by internal fractures in high strength steels. Research on gigacycle fatigue first needed an accelerated fatigue testing method. Ultrasonic fatigue testing at 20 kHz was then a powerful tool, since using a test frequency of 20 kHz allows 10^9^ cycles to be completed in one day, unlike the 3–4 months needed for conventional fatigue testing. Although the frequency effect was a potential concern, it proved negligible under conditions in which internal fractures occurred. Ultrasonic fatigue testing is therefore concluded to be effective for researching gigacycle fatigue that is caused by internal fractures.

Next, gigacycle fatigue tests were conducted on various materials and under various conditions. The results of these experiments revealed numerous unique characteristics of internal fractures. For example, although the fatigue strengths of conventional surface fractures were closely dependent on tensile strength, the influence of tensile strength on internal fractures was very minor. Internal fractures were instead affected by the size of inclusions at fracture origins. Other than this, differences were also reported in hydrogen effects, mean stress effects and size effects. The effects of hydrogen were dramatically greater on internal fractures than on conventional surface fractures. The mean stress effects were more serious in titanium alloys than in high strength steels. Ti-6Al-4V alloys showed significant degradation of gigacycle fatigue strength under tensile mean stress conditions, and as a result, their modified Goodman lines strayed into the danger area. However, this pattern was not observed in high strength steels. In contrast, size effects were notable in high strength steels: large specimens showed clearly lower fatigue strengths, revealing larger inclusions at the internal fracture origins.

These special characteristics of internal fractures indicated the need for a unique model to predict gigacycle fatigue strength. Accordingly, elucidation of the mechanisms involved was required to reveal which process controlled the internal fractures: crack initiation, crack growth, or non-propagating cracks. Crack growth appeared the most likely, but the drawback in this case was the adequacy of the calculated internal crack growth rates. Extremely slow crack growth rates, which were much smaller than the lattice length, were calculated for gigacycle fatigue. To investigate this phenomenon, the author attempted to measure the crack growth rates of these small internal cracks using a beach mark method. The results showed extremely slow crack growth rates for small internal cracks, indicating that the growth of these small internal cracks controlled the internal fractures. The fatigue life was thus very close to the crack growth life of these small internal cracks.

Crack growth life was calculated using fracture mechanics. However, when a conventional crack growth law was applied to small internal cracks, the predicted fatigue life curve proved highly implausible, as it markedly overestimated the effects of inclusion size. To correct this problem, a new model was proposed, using a new crack growth law specialized for small internal cracks. This new model predicted more realistic fatigue life curves. Finally, predictions based on the new model were derived for several grades of high strength steels. The predicted gigacycle fatigue strengths revealed only minor differences between steel grades. The decrease in fatigue strengths became less steep as a function of rising inclusion size, resulting in an estimated fatigue strength of 500 MPa at 10^9^ cycles, even for 100-µm inclusions. The mean stress effects were safely evaluated by the modified Goodman line without seeing it enter the danger zone, as seen in Ti-6Al-4V alloys.

As discussed in this review, research on the gigacycle fatigue of high strength steels has progressed rapidly in the last one or two decades; however, there is room for more study. For example, although a great deal of research data has been acquired for oxide-type inclusions, we have less information on other types of inclusions, such as sulfides. This could be a good topic for a future study.
